# The Rheumatoid Arthritis Risk Gene AIRE Is Induced by Cytokines in Fibroblast-Like Synoviocytes and Augments the Pro-inflammatory Response

**DOI:** 10.3389/fimmu.2019.01384

**Published:** 2019-06-18

**Authors:** Beatrice Bergström, Christina Lundqvist, Georgios K. Vasileiadis, Hans Carlsten, Olov Ekwall, Anna-Karin H. Ekwall

**Affiliations:** ^1^Department of Rheumatology and Inflammation Research, Institute of Medicine, The Sahlgrenska Academy, University of Gothenburg, Gothenburg, Sweden; ^2^Centre for Bone and Arthritis Research, The Sahlgrenska Academy, University of Gothenburg, Gothenburg, Sweden; ^3^Department of Pediatrics, Institute of Clinical Sciences, The Sahlgrenska Academy, University of Gothenburg, Gothenburg, Sweden

**Keywords:** rheumatoid arthritis, fibroblast-like synoviocytes, inflammation, cytokines, AIRE, interferon response genes

## Abstract

The autoimmune regulator AIRE controls the negative selection of self-reactive T-cells as well as the induction of regulatory T-cells in the thymus by mastering the transcription and presentation of tissue restricted antigens (TRAs) in thymic cells. However, extrathymic AIRE expression of hitherto unknown clinical significance has also been reported. Genetic polymorphisms of *AIRE* have been associated with rheumatoid arthritis (RA), but no specific disease-mediating mechanism has been identified. Rheumatoid arthritis is characterized by a systemic immune activation and arthritis. Activated fibroblast-like synoviocytes (FLS) are key effector cells, mediating persistent inflammation, and destruction of joints. In this study, we identified AIRE as a cytokine-induced RA risk gene in RA FLS and explored its role in these pathogenic stroma cells. Using RNA interference and RNA sequencing we show that AIRE does not induce TRAs in FLS, but augments the pro-inflammatory response induced by tumor necrosis factor and interleukin-1β by promoting the transcription of a set of genes associated with systemic autoimmune disease and annotated as interferon-γ regulated genes. In particular, AIRE promoted the production and secretion of a set of chemokines, amongst them CXCL10, which have been associated with disease activity in RA. Finally, we demonstrate that AIRE is expressed in podoplanin positive FLS in the lining layer of synovial tissue from RA patients. These findings support a novel pro-inflammatory role of AIRE at peripheral inflammatory sites and provide a potential pathological mechanism for its association with RA.

## Introduction

Rheumatoid arthritis (RA) is a systemic inflammatory autoimmune disease pre-dominantly affecting joints (1). If left untreated, the disease progresses to tissue destruction, functional disability, and comorbidities such as cardiovascular disease. RA most likely evolves over many years as a consequence of repeated environmental stress, causing inflammatory events, and immune activation and eventually breakdown of tolerance, in genetically pre-disposed individuals (2). In the joints, the disease is characterized by persistent inflammation and formation of a hyperplastic invasive synovium. Key players in these processes are activated fibroblast-like synoviocytes (FLS), which possess tumor cell-like features such as increased cell proliferation and the ability to invade and destroy surrounding tissue (3). The pro-inflammatory cytokines tumor necrosis factor (TNF) and interleukin-1β (IL-1β) are typical activators of FLS, inducing production of e.g., pro-inflammatory molecules and matrix degrading enzymes (4, 5). In addition, in response to interferon-γ (IFN-γ) RA FLS up-regulate the expression of major histocompatibility complex (MHC) class II, which suggests a role in antigen presentation and direct interaction with immune cells (6). Once activated, RA FLS continue their aggressive tissue destructive behavior without the need of further stimulation from the immune system. This might contribute to the fact that only 20–40% of RA patients achieve sustained clinical remission by the currently available immunosuppressive anti-rheumatic therapies (7). It also demonstrates the urgent need for novel drugs targeting the RA FLS.

Today, more than 100 RA risk genes have been identified by analyses of single nucleotide polymorphisms in Genome Wide Association Studies (GWAS) (8, 9). Several GWA studies have demonstrated that polymorphisms (rs2075876, rs760426, rs878081) in the autoimmune regulator gene, *AIRE*, are associated with RA (10–12). Most studies on the mechanisms by which the RA-associated genetic variants influence disease have focused on immune cells. Interestingly, integrative analysis of multiple omics data including epigenetic marks from RA FLS and controls has identified AIRE as one of seven candidate genes for the pathogenic features of RA FLS (13). We have earlier demonstrated that *LBH*, another of these seven genes, regulates cell cycle progression in FLS (14, 15). The potential role of AIRE in FLS has not been investigated.

AIRE is a master regulator of the transcription of tissue restricted antigens (TRAs) in medullary thymic epithelial cells (mTEC) (16). The induced TRAs are subsequently presented on MHC class II for maturing thymocytes, and self-reactive thymocytes are either deleted or diverted into natural regulatory T cells (nTregs) as a central part of the T-cell tolerance induction in the thymus. The importance of this process is illustrated by the autoimmune polyendocrine syndrome type-1 in which mutations in *AIRE* result in multiple severe autoimmune manifestations (17). Interestingly, RA is not a feature of APS1 and only a few cases of arthritis has been described in APS1 patients. *AIRE* expression in mTEC is induced by RANKL and TNF, and tightly regulated by complex molecular mechanisms involving epigenetic modifications (18).

Apart from the well-established expression and function of *AIRE* in mTECs, *AIRE* expression in thymic B-cells and extra-thymic expression in lymph nodes and in keratinocytes has been described (19). Interestingly, as opposed to the tolerogenic role of AIRE in the thymus and lymph nodes, AIRE has been reported to interact with keratin 17 and induce the expression of pro-inflammatory genes, most notably *CXCL9, CXCL10*, and *CXCL11*, in keratinocytes in acute inflammation and tumorigenesis (20).

In this study, we investigated the transcriptome of TNF and IL-1β-activated RA FLS and identified AIRE as one of 24 differentially expressed RA risk genes in these cells. We demonstrate that AIRE is expressed in the RA synovium and augments the inflammatory response by promoting the expression of an interferon γ signature including the production of chemokines in FLS.

## Materials and Methods

### Biological Samples

Human synovial tissue specimens were obtained from patients with RA or osteoarthritis (OA) during joint replacement surgery at Sahlgrenska University Hospital and Spenshult Hospital in Sweden. All RA patients fulfilled the American College of Rheumatology 1987 revised criteria for the disease (21). Human thymic tissue was obtained from children undergoing corrective cardiac surgery at Sahlgrenska University Hospital, Gothenburg, Sweden. The procedures were approved by the Ethics Committee of Gothenburg and all patients gave written informed consent. Homogenous cultures of primary FLS were established as described earlier (22) and used in passage 4–8.

### Cell Culture and Stimulations

Primary FLS were cultured in Dulbecco's modified Eagle's medium (DMEM) GlutaMAX (Gibco, Carlsbad, CA, USA) supplemented with antibiotics (penicillin/streptomycin, gentamicin) and 10% heat-inactivated fetal bovine serum (FBS), in a humidified 5% CO_2_ and 37°C atmosphere. Primary normal neonatal human dermal fibroblasts (HDFn) were obtained from ATCC (PCS-201-010). For stimulation experiments, cells were seeded into 6-well-plates (qPCR, flow cytometry, RNAseq) or 8-well-chamber slides (immunofluorescence) and incubated until confluence. The cells were serum-starved for 12 h in 1% FBS and then stimulated with the human recombinant proteins IL-1β (R&D Systems, Minneapolis, MN, USA), TNF (Invitrogen, Carlsbad, CA, USA), or RANKL (R&D Systems) for 12–24 h.

### RNA Extraction and Gene Expression Analysis

Total RNA was isolated from cells using RNeasy Micro Kit (Qiagen) according to the manufacturer's instructions and quantified with a NanoDrop 100 spectrophotometer (Thermo Scientific). Complementary DNA (cDNA) was synthesized from isolated RNA using TaqMan reverse transcription agents (Applied Biosystems, Carlsbad, CA, USA). qPCR was performed on a ViiA 7 Real-Time PCR System, using TaqMan reagents, and pre-designed primer-probe sets (Table S1) from Applied Biosystems. Ct values were normalized to glyceraldehyde 3-phosphate dehydrogenase (GAPDH) expression and fold change in mRNA expression was calculated using the ΔΔct-method (2^ΔΔCt^).

### Immunofluorescence and Confocal Microscopy

Paraformaldehyde fixed paraffin-embedded tissue sections were rehydrated and subjected to antigen retrieval in a pressure chamber (2100 Retriever, Aptum Biologics Ltd., Southampton, UK). Unspecific binding was blocked using serum-free protein block (DAKO, Glostrup, Denmark) supplemented with 5% normal donkey serum (D9663, Sigma-Aldrich, Saint Louis, Mo, USA). Incubation with goat anti-human AIRE antibody and/or mouse anti-human podoplanin for 60 min in RT followed by secondary Alexa Fluor labeled antibodies (Table S2). Thresholds of positive signal in the confocal microscopy were set using normal goat serum (S-1000, Vector Laboratories, Burlingame, CA, USA) or normal rabbit serum (DAKO, Glostrup, Denmark) followed by the secondary antibody.

### Flow Cytometry and ImageStream X

Cells were fixed with Foxp3 transcription factor fixation/permeabilization kit (00-5521-00, eBioscience) and blocked using Beriglobin (CSL Behring L, PA, USA). The cells were stained in 100 μL with antibodies and dilutions as shown in Table S2. For biotinylated antibodies, samples were stained with Streptavidin-Alexa Fluor 647 (S32357, Life Technologies). Before acquisition on a FACSVerse (BD Biosciences, San Jose, CA, USA) nuclear stain Hoechst 33342 (H3570, Life Technologies) was added to a concentration of 0.5 μg/ml. Data were analyzed using FlowJo software (TreeStar Inc., Ashland, OR, USA). For single cell imaging, cells were acquired and analyzed on an ImageStream X Mark II imaging flow cytometer (Amnis, Seattle, WA, USA). CXCL10 levels in undiluted cell culture supernatants were assessed using LEGENDplex reagents (BioLegend, San Diego, CA, USA) according to the manufacturer's instructions.

### Gene Silencing

Small interfering RNA (siRNA) targeting human AIRE (#L-010993-00) and non-targeting (NT) control (#D-001810-10, Thermo Scientific Dharmacon, Lafayette, CO, USA) were transfected into primary FLS using Amaxa Nucleofector Technology and HDF Nucleofector solution (Lonza, Basel, Switzerland). The cells were plated in 6-well plates and incubated for 2 days prior to serum starvation and stimulation. One NT sample of each line was unstimulated (“no AIRE”) and the other NT control and the AIRE silenced sample were stimulated with IL-1β+TNF for 24 h generating “high AIRE” and “low AIRE” samples. Samples were collected in RLT buffer and RNA isolated as described in Methods. The efficiency of gene silencing was assessed by qPCR and RNAseq.

### RNA Sequencing and Bioinformatics Analysis

RNA sequencing was performed using the Nextseq500 plattform, 2 × 75 read length and Nextseq500 Kit High Output V2 reagents. The library was prepared using TruSeq stranded Total RNA Sample preparation kit with Zero Gold according to the preparation guide (15031048 Rev. E). A quality assessment was performed on the data using FastQC (https://www.bioinformatics.babraham.ac.uk/projects/fastqc/). The fastq files were filtered with prinseq (version 0.20.3). The quality filtered fastq files were mapped toward the human reference genome (hg19, UCSC assembly, February 2009) with STAR (version 2.5.2b). The alignment was sorted and indexed with SAM tools (version 1.3.1). Htseq (version 0.5.3p3) was used for calculation of the gene counts. Differentially expressed genes were identified with DEseq2 using Benjamini-Hochberg *p*-value adjustment (23). The identified genes were grouped into TRAs. The annotation of TRA was downloaded from BioGPS using the dataset GSE1133 (GeneAtlas U133A, gcrma). A pathway analysis was performed on the differentially expressed genes with IPA (Ingenuity® Systems, http://www.ingenuity.com/). The Gene Set Enrichment Analysis (GSEA) was performed as follows; all genes from the comparison STIM NTC vs. STIM AIRE KD were ranked based on fold change. The gene sets of Hallmark Inflammatory Response and Hallmark Interferon Gamma Response in gene matrix transposed file formats (gmt) were downloaded from molecular signature database [(24) http://www.broad.mit.edu/gsea/]. The gene set analysis itself was run as GSEA pre-ranked for 1,000 permutations.

### Statistical Analysis

Statistical analysis of gene expression was performed on Δct values. Gaussian distribution was confirmed by Shapiro-Wilk's normality test followed by paired or unpaired two-tailed *t*-test as indicated. Graphs display mean with SEM and dots represent individual values. One-way ANOVA with Dunnett's multiple comparisons test was used for multiple tests to the unstimulated control. *P* ≤ 0.05 was considered to be significant. Differentially expressed genes were considered significant if adjusted *p* ≤ 0.05. Bioinformatics analysis of RNA seq data as stated above.

## Results

### Pro-inflammatory Cytokines Modify Expression of RA Risk Genes in RA FLS

**Figure 1:**
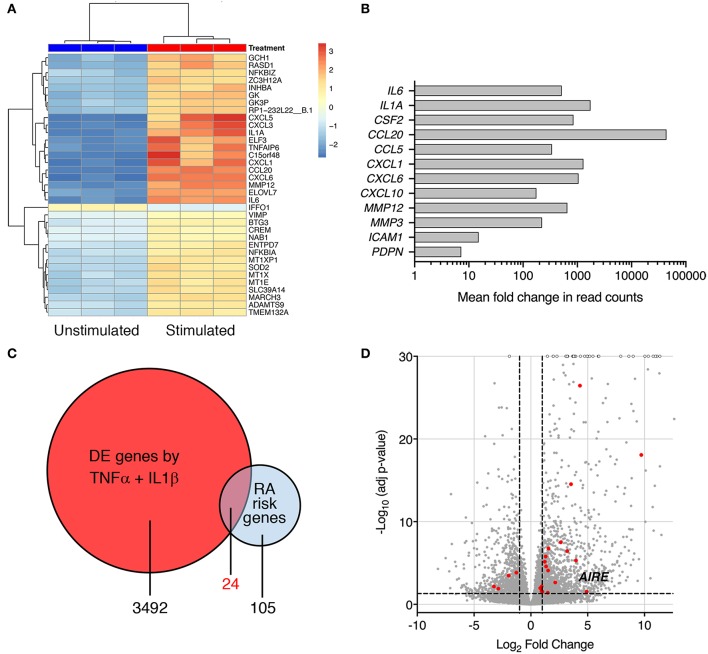
Pro-inflammatory cytokines modify expression of RA risk genes in RA FLS. **(A)** A heatmap with hierarchical clustering of the top 35 significantly (Adj *P* < 0.05) differently expressed genes by RNA seq in IL-1β (2 ng/ml) + TNF (5 ng/ml) stimulated vs. unstimulated RA FLS from three different patients. **(B)** Increased expression of genes characterizing the “activated” aggressive RA FLS phenotype: pro-inflammatory cytokines (IL-6, IL-1A, GM-CSF), chemokines (CCL20, CCL5, CXCL1, CXCL6, and CXCL10), matrix degrading enzymes (MMP12 and 3) and adhesion molecules (ICAM-1, podoplanin) in IL-1β + TNF stimulated vs. unstimulated RA FLS by RNAseq (Log2 fold change in normalized read counts, *n* = 3). **(C)** Venn diagram of the RA-associated risk genes by GWAS and TNF + IL-1β induced DE genes by RNAseq. **(D)** Volcano plot (each dot representing one gene plotted based on log2 fold change and –log10 of adj *p*-value by DEseq2) of the TNF + IL-1β induced DE genes with the DE RA risk genes, including *AIRE*, highlighted in RED. Dashed lines at adjusted *p* = 0.05 and at fold change = ±2.

Interleukin-1β and TNF are abundant cytokines in the RA joint and known inducers of an “activated” RA FLS phenotype. In the search for novel important pathways in RA, we investigated the transcriptome of *cytokine-activated* compared to untreated control RA FLS using RNA sequencing. The combined IL-1β (2 g/ml) + TNF (5 ng/ml) stimulation induced a highly significant transcription of genes known to shape the aggressive phenotype of RA FLS. In total, more than 3,400 genes were significantly differentially expressed (adj. *p* < 0.05) by stimulation compared with unstimulated RA FLS (Top 30 in Figure 1A), illustrating the impact of these cytokines on the cells. In particular, a strong induction was seen on cytokines, chemokines, matrix degrading enzymes, and pro-migratory molecules with adjusted *p*-values in the range 10^−8^ to 10^−62^, *n* = 3 (Figure 1B). The FLS also changed morphology, displaying a dendritic phenotype different from the unstimulated polygonal spread shape (not shown). Interestingly, we found differential expression (adj. *p* < 0.05) of 24 of the 105 known RA risk genes (9) in the TNF + IL-1β stimulated RA FLS compared to unstimulated (Figure 1C, Table S3). Most of them (17 genes) were up-regulated ≥2 fold (Figure 1D) and the autoimmune regulator, *AIRE*, was one of them (fold change = 30.1, Adj *p* = 0.030).

### Cytokine-Induced *AIRE* Expression Is Higher in RA Compared to Control FLS

**Figure 2:**
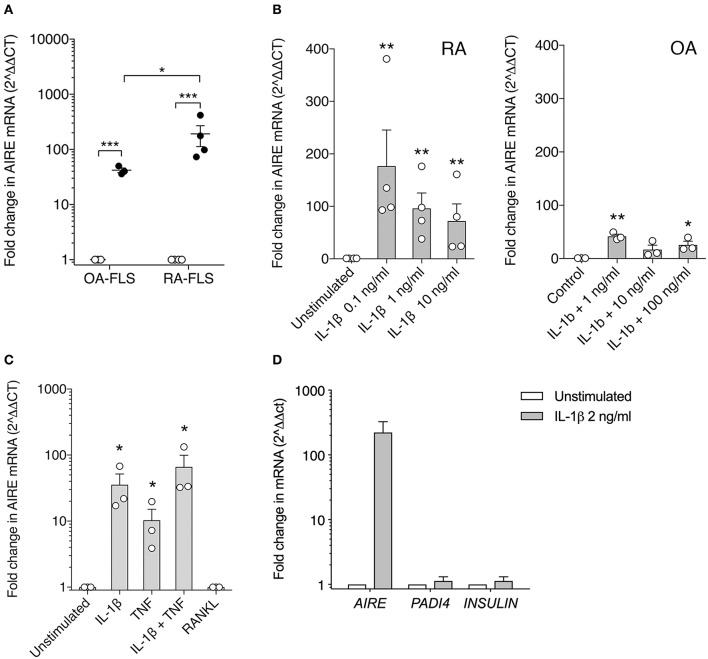
AIRE mRNA expression is induced in FLS by pro-inflammatory cytokines. **(A)** AIRE mRNA expression is significantly increased by IL-1β 1 ng/ml (filled circles) compared to unstimulated (open circles) in both OA (42 ± 4 fold, *n* = 3, *p* = 0.0006) and RA FLS (191 ± 79 fold, *n* = 4, *p* = 0.001). Statistics with paired two-tailed *t*-test. The IL-1β -induced AIRE expression was significantly higher in RA compared to OA FLS (*p* = 0.038, unpaired two-tailed *t*-test). **(B)** Dose response of IL-1β stimulation on AIRE mRNA expression in RA FLS (*n* = 4, *p* = 0.0001) and OA FLS (*n* = 3, *p* < 0.0001) One-way ANOVA and Dunnett's test. **(C)** Effects of the FLS activating cytokines IL-1β (2 ng/ml) and TNF (5 ng/ml) and combined compared to unstimulated in OA-FLS (*n* = 3). One-way ANOVA and Dunnett's. RANKL (5 ng/ml) did not induce AIRE mRNA in FLS. **(D)** The tissue specific antigens; PADI4 and INSULIN mRNA are not induced in IL-1 β stimulated AIRE expressing FLS by qPCR (Fold change in stim vs. unstim, *n* = 3 FLS). ^*^p ≤ 0.05, ^**^p ≤ 0.01 and ^**^p ≤ 0.001.

To confirm this finding, we investigated AIRE expression in primary human FLS using qPCR. We did not detect any *AIRE* mRNA expression in unstimulated FLS from RA or control OA patients (Figure 2A). However, *AIRE* mRNA was strikingly induced, up to 191 ± 79 fold, by IL-1β in RA FLS (*p* = 0.001, *n* = 4) and 42 ± 4 fold *(p* = 0.0006, *n* = 3) in OA FLS (Figure 2A) after 24 h. The *AIRE* induction was significantly higher in RA than OA FLS (*p* = 0.035). Isolated RNA from human thymic epithelial cells was used as positive control and the *AIRE* mRNA expression in mTEC was 24-fold higher than average expression in RA FLS stimulated with IL-1β (data not shown). IL-1β 0.1–1 ng/ml was the most efficient dose within the bioactive range in FLS and the interindividual variations were greater in RA compared with OA FLS (Figure 2B). Furthermore, we found that *AIRE* was also induced 10 ± 5 fold (*p* = 0.022) by TNF compared to unstimulated in OA FLS and the largest effect was seen using IL-1β + TNF (66 ± 33 fold, *p* = 0.018) (Figure 2C). RANKL, the main inducer of AIRE expression in the thymus, did not induce *AIRE* mRNA in FLS (Figure 2C).

In the thymus, AIRE induces expression of hundreds of TRA in mTEC cells for tolerance purposes. However, no induction of the known AIRE-regulated genes, *INS* (insulin), and *PADI4* (peptidyl arginine deiminase type 4), were detected in the IL-1β stimulated AIRE expressing FLS (Figure 2D).

### Nuclear AIRE Expression Is Present in TNF + IL-1β Stimulated RA FLS

**Figure 3:**
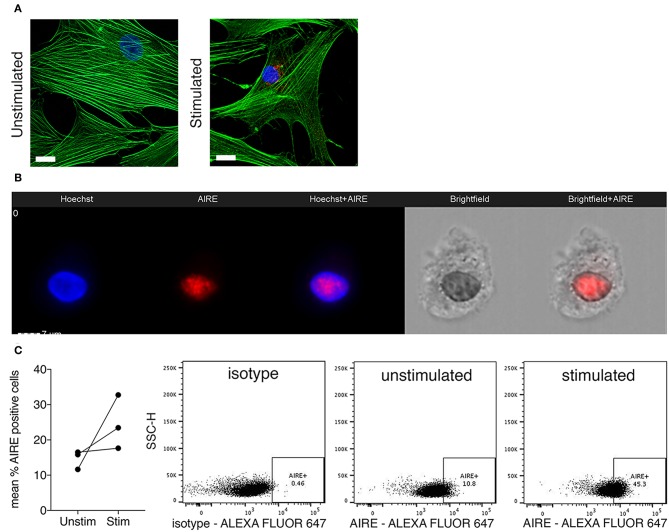
Nuclear AIRE expression is present in cytokine stimulated RA FLS. **(A)** Representative immunofluorescence image of AIRE expression (red) in IL-1β +TNF stimulated but not in unstimulated RA FLS. Actin in green, nuclei in blue. Bar 5 μm. **(B)** Representative images of nuclear AIRE expression (red) in one IL-1β +TNF stimulated RA FLS using ImageStreamX flow cytometry. Nuclei in blue (Hoeschst). Merged Hoeschst + AIRE in the mid panel. Brightfield images to the right. **(C)** The mean AIRE protein expression (% positive cells) in unstimulated vs. IL-1β+TNF stimulated RA FLS (*n* = 3, two experiments) by flow cytometry. Representative dotplots of isotype, unstimulated, and IL-1 β +TNF stimulated samples.

Next, we investigated if AIRE is expressed also on the protein level in cytokine stimulated primary human FLS. A speckled nuclear, but also perinuclear, AIRE protein expression was detected using confocal immunofluorescence in a small fraction of IL-1β + TNF stimulated, but not in unstimulated RA FLS cultured in monolayer (Figure 3A). To further investigate the subcellular localization of AIRE in FLS we performed ImageStreamX flow cytometry on stimulated FLS and found that AIRE localized to the nuclei with the same speckled pattern as seen on tissue sections of RA synovium and thymus (Figure 3B). Using flow cytometry, the mean AIRE expression from two different experiments was increased by 68% (*n* = 3 per experiment) by IL-1β + TNF stimulation (Figure 3C). In this experimental setting, there was slightly lower fluorescence intensity in the isotype control compared to unstimulated control (possibly due to antibody/fluorochrome properties).

### AIRE Does Not Enrich for TRA Expression in Cytokine Activated RA FLS

**Figure 4:**
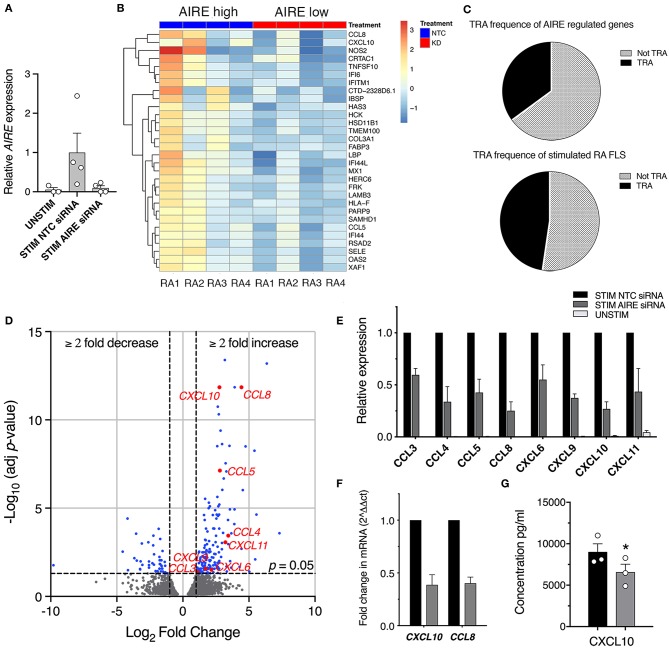
AIRE regulates transcription of pro-inflammatory genes in activated RA FLS. **(A)** Relative *AIRE* expression in unstimulated, stimulated NTC, and AIRE silenced (KD) samples by normalized counts by size factor (*n* = 4 different RA FLS lines), **(B)** Heat map with hierarchical clustering of the top 30 of in total 217 significantly (Adj *P* < 0.05) DE genes by RNA seq in AIRE high vs. low samples. **(C)** Frequency of TRA (black) and not TRA (gray pattern) in stimulated RA FLS based on available data on tissue expression in the BioGPS database. Upper graph represents the 191 annotated AIRE regulated genes. Lower graph, the mean of three random sets of 191 annotated genes in the RNAseq dataset. **(D)** Volcano plot showing the 217 DE by in AIRE high compared to AIRE low samples (BLUE dots) and chemokines highlighted in RED. Dashed lines at adjusted *p* = 0.05 and at fold change = ±2. **(E)** Relative expression of chemokine genes in AIRE high vs. AIRE low samples normalized counts by size factor and **(F)** Relative expression of CXCL10 and CCL8 by qPCR in AIRE high vs. AIRE low samples (*n* = 3 transfected RA FLS lines). **(G)** Concentration of CXCL10 in the supernatant of TNF + IL-1β-stimulated transfected AIRE high (NTC) compared to AIRE low (AIRE siRNA) RA FLS (*p* = 0.026, *n* = 3 different RA FLS lines) by bead-based flow cytometric immunoassay. Statistics with paired two-tailed *t*-test. ^*^p ≤ 0.05.

While we have extensive knowledge of the functional consequences of AIRE expression in mTECs, the role of AIRE in other cell types is largely unknown. To explore the functional role of AIRE in FLS, we performed *AIRE* gene silencing using primary RA FLS cultures from four patients. The transfected cells were stimulated with 5 ng/ml TNF + 2 ng/ml IL-1β or kept in medium with 1% FBS for 24 h; generating samples of (1) no AIRE (unstimulated), (2) high AIRE (stimulated, non-target control siRNA), and (3) low AIRE expression (stimulated, *AIRE* siRNA); followed by RNA sequencing. The mean silencing efficiency was 71% as quantified by qPCR (3 samples had ≥84% knock-down) and the mean induction of *AIRE* in the stimulated samples was 20-fold (by normalized read counts) compared with unstimulated. However, the induction of AIRE was 12.5-fold different between the lowest and the highest expression illustrating the biologic variation in the patient samples (Figure 4A, see also Figure 2B, RA). Subsequent bioinformatic analysis identified in total 217 differentially expressed (DE) genes in stimulated “high AIRE” compared to stimulated “low AIRE” samples (adjusted *p* < 0.05) of which 171 genes were increased (up to 159-fold) and 46 decreased (down to 0.001). A heat map with the top 30 DE genes to the mean (adjusted *p* < 0.05) is shown in Figure 4B.

Based on the described function of AIRE in mTEC we first investigated if the induced genes were enriched for TRAs based on expression data from the BioGPS-database (www.biogps.org) and by defining TRAs as genes with an expression restricted to up to five tissues (25). However, of the 191 annotated (with available tissue expression data) AIRE regulated genes, there was no enrichment of TRAs (35%) compared to non-AIRE regulated genes (48% TRA; mean of three random set of 191 genes in the data set using R statistics software) (Figure 4C, Table S4). As mentioned earlier, a functional up-regulation of MHC class II on RA FLS has been demonstrated *in vitro* in response to interferon γ. In addition, MHC class II expression on FLS has been demonstrated in synovial biopsies from RA joints (26). However, we did not find any significant increase of the MHC class II trans-activator gene (CIITA) or the HLA genes encoding MHC class II proteins in the “High AIRE” samples (data not shown), further indicating that AIRE might have another role in FLS than inducing and presenting TRAs for tolerance induction purposes as in mTECs.

### AIRE Augments Expression of Pro-inflammatory Genes in Activated FLS

Interestingly, the expression of eight chemokines (*CXCL10, CCL8, CCL5, CCL4, CXCL11, CXCL9, CXCL6*, and *CCL3*) were significantly higher in the High compared to Low AIRE samples (Figure 4D), indicating that AIRE might be regulating parts of the pro-inflammatory response induced by TNF and IL-1β. In particular, the monocyte chemoattractant protein 2 (MCP-2 or *CCL8*) was increased 21.6-fold (*p* = 1.4 · 10^−12^) and interferon γ-induced protein 10 (IP-10 or *CXCL10*) 6.8-fold (*p* = 1.4 · 10^−12^) (Figure 4E). Unstimulated FLS did not express any significant levels of these eight chemokines (Figure 4E). A reduced mRNA expression of *CXCL10* and *CCL8* in AIRE-silenced samples was confirmed by qPCR in RA FLS (Figure 4F).

In order to validate this, we measured the concentration of CXCL10 in the supernatants of transfected AIRE High and AIRE Low RA FLS using bead-based flow cytometric immunoassay. CXCL10 was significantly lower in AIRE silenced compared to AIRE high samples (6,548 ± 978.5 vs. 8,980 ± 1,008 pg/ml, *p* = 0.026, *n* = 3 RA FLS lines) (Figure 4G).

### AIRE Masters Expression of an Interferon-γ Signature in Activated FLS

**Figure 5:**
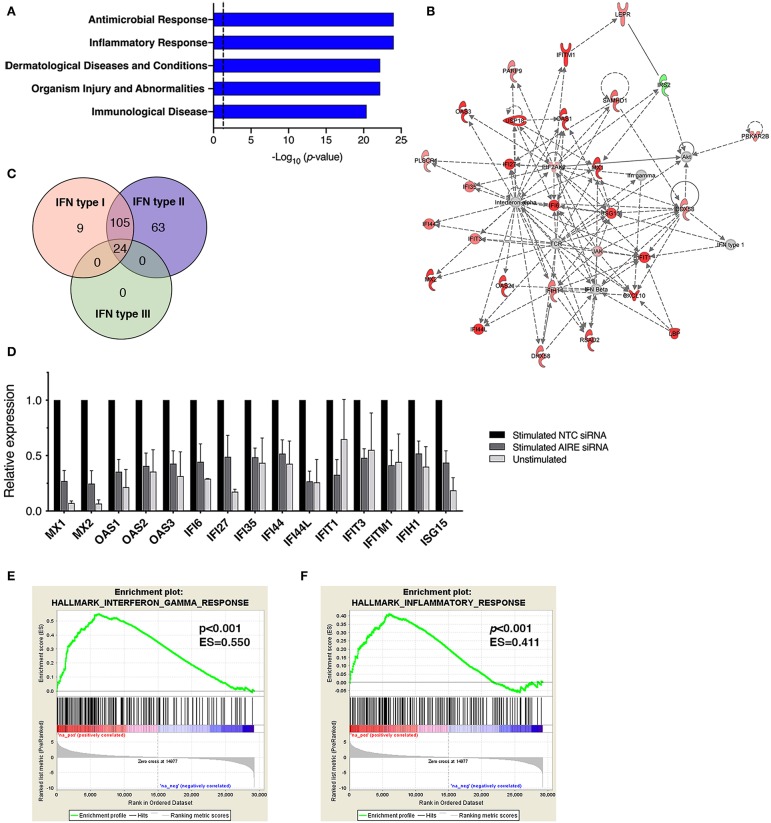
AIRE masters expression of an interferon-γ signature in activated FLS. **(A)** The top five canonical pathways of an unbiased Ingenuity Pathway Analysis (IPA) of the DE AIRE high compared to AIRE low data set (*n* = 4 different RA FLS lines) using adjusted *p* < 0.05 and differential expression ≥±1.0 log2 fold change as cut off levels. **(B)** The most significant network: “Antimicrobial response” with differentially expressed genes in AIRE high vs. AIRE low samples by IPA. Increased expression in RED and reduced expression in GREEN symbols. **(C)** Classification of the 201 (93%) AIRE regulated genes annotated as interferon regulated genes (IRG) by the INTERFEROME v2.01 database, showing a pre-dominance (96%) of IFN-γ (type II) regulated genes. **(D)** Relative expression (based on normalized counts by size factor) of AIRE-dependent IRGs in unstimulated (no AIRE) and stimulated AIRE low samples compared to AIRE high samples in the RNA seq data. **(E)** Gene set enrichment analysis (GSEA) of the *Hallmark Interferon Gamma Response* gene set in the DE AIRE high compared to AIRE low data set showing an enrichment score (ES) of 0.550 (*p* < 0.001) for AIRE-dependent genes in this gene set and an ES of 0.411 (*p* < 0.001) of the *Hallmark Inflammatory Response gene* set in **(F)**.

In addition to CXCL10 and CCL5, we found that several other genes associated with inflammation of skin (active lesions) in psoriasis (27, 28) were among the top DE genes by AIRE in RA FLS; *NOS2* (iNOS), *MX1, OAS2, IFI44, MX2*, and *OAS1*. And indeed, an unbiased Ingenuity Pathway Analysis (IPA) on the High vs. Low AIRE samples using adjusted *p* < 0.05 and differential expression ≥±1.0 log_2_ fold change as cut off levels, identified “Antimicrobial response” and “Inflammatory response” (*p* = 9.5 · 10^−25^) as well as “Dermatological Diseases and Conditions” and “Organism Injury and Abnormalities” (*p* = 6.6 · 10^−23^) as the most significant pathways (Figure 5A) with antiviral response and psoriasis as top diseases. “Antimicrobial Response” was the most significant network with a score of 46 (Table 1 and Figure 5B).

**Table 1:** Pathway analysis using Ingenuity Pathway Analysis of differentially expressed genes (Adjusted *p* <0.05 and differential expression ≥±1.0 log^2^ fold change) in “High AIRE” vs. “Low AIRE” samples identified the following top significant network.

**Number**	**Molecules in Network**	**Score**	**Focus molecules**	**Top functions**
1	Akt, CXCL10, DDX58, DHX58, EIF2AK2, IFI27, IFI35, IFI44, IFI44L, IFI6, IFIH1, IFIT1, IFIT3, IFITM1, IFN Beta, IRS2, ISG15, IFN gamma, IFN alpha, JAK, LBP, LEPR, MX1, MX2, OAS1, OAS2, OAS3, PARP9, PLSCR1, PRKAR2B, RSAD2, SAMHD1, TCR, USP18	46	28	Antimicrobial response, Inflammatory response

A list of predicted upstream regulators of target molecules in the dataset from the IPA prompted us to investigate if the AIRE induced genes were involved in interferon signaling. Using the Interferome v2.01 bioinformatics database of interferon responsive genes (www.interferome.org) we concluded that 96% of the annotated AIRE regulated genes were classified as interferon response genes (IRGs) with a pre-dominance (95.5%) of IFNγ (type II) IRGs (Figure 5C). Interestingly, IL-1β + TNF stimulation of RA FLS did not induce IFNγ mRNA (RNA seq data set) or secretion of IFNγ to the supernatant of the cell cultures (assessed by ELISA, data not shown) suggesting that AIRE induces the interferon signature independent of IFNγ. Relative expressions of the DE IRGs are displayed in Figure 5D. To test the hypothesis that AIRE promotes the expression of a pro-inflammatory IFNγ signature we performed GSEA of the gene set Hallmark Interferon Gamma Response on the AIRE High vs. AIRE Low data set which demonstrated an enrichment score (ES) of 0.550 (*p* < 0.001) and a negative ES of 2.126. *MX2* had the highest rank (5.567) and *STAT2* the highest running ES (Figure 5E). Running analysis of the Hallmark Inflammatory response gene set gave an ES of 0.411 (*p* < 0.001) and NES of 1.562 (Figure 5F). In both gene sets, the anti-inflammatory Interleukin-10 receptor α gene (*IL10RA*) had the lowest rank (-4.157) further supporting a pro-inflammatory role of AIRE.

### AIRE Is Expressed in Activated FLS of the Synovial Lining Layer in RA

**Figure 6:**
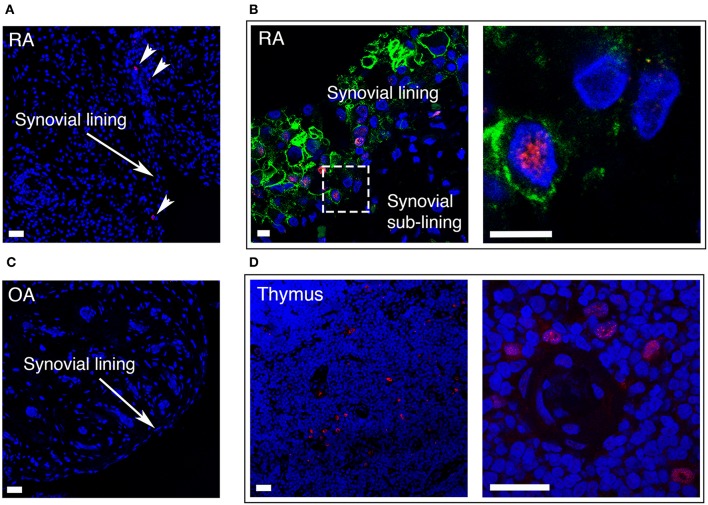
AIRE is expressed in the synovial lining layer in RA. **(A)** RA synovium stained with S.C. anti-AIRE antibody (RED). Arrow heads show positive cells in the synovial lining layer. Bar 20 μm. **(B)** RA synovium double stained with antibodies toward the surface marker podoplanin (GREEN) of “activated” lining layer FLS and AIRE (RED, Abcam ab). Note the increased thickness of the lining layer in RA compared to OA in Figure 1C. Encircled area enlarged in the panel to the right showing an AIRE expressing activated FLS. Bar 5 μm. **(C)** OA synovium stained negative with S.C. anti-AIRE antibody. Bar 20 μm. **(D)** Positive control staining of human thymus tissue stained with S.C. anti-AIRE antibody (RED). Overview of the thymic medulla in the left panel and an area surrounding an Hassall's corpuscle with AIRE positive mTECs enlarged in the right panel. Bar 20 μm. Nuclei with Hoeschst (BLUE).

In RA, the number of FLS in the synovial lining layer increases and activated RA FLS play a key role in the formation of the pannus tissue. We performed immunofluorescence staining of synovial tissue samples from RA patients (*n* = 5), and found that AIRE expressing cells were present in the lining layer of the RA synovium (Figure 6A). The pro-invasive glycoprotein podoplanin is up-regulated on lining layer FLS in RA and by TNF and IL-1β *in vitro* (22). Double staining with AIRE and podoplanin demonstrated intense podoplanin staining in areas with AIRE positive cells including double positive cells (Figure 6B) indicating that AIRE is expressed in areas of FLS activation. We did not find AIRE expression in control tissue from patients with OA (*n* = 4) (Figure 6C). We confirmed AIRE reactivity of the antibodies on human thymic sections where a similar speckled staining pattern of mTEC nuclei was evident (Figure 6D).

## Discussion

More than 100 RA-associated risk genes have been identified, but for the majority of those, the mechanisms for disease susceptibility are unknown. It has been reported that genetic variations of *AIRE* pre-dispose for RA, and multiple omics analysis (13) have pointed to a role for this autoimmune regulator in FLS. Our study demonstrates that as many as 24 RA risk genes, including *AIRE*, are differentially expressed in RA FLS stimulated by TNF and IL-1β. In particular, AIRE is not expressed in unstimulated FLS but induced upon activation by these pro-inflammatory cytokines.

Interestingly, AIRE was also induced by TNF + IL-1β in OA FLS and in dermal fibroblast *in vitro*, but to a significantly lower level and with less variability than in RA FLS. It has been shown that RA FLS are primed to enhanced pathogenic responses by repeated or persistent cytokine stimulation *in vitro* (29). This together with genetic and epigenetic factors might explain the enhanced expression of AIRE in RA, which alone or in concert with other factors of the RA joint may have pathogenic impact on disease development and activity.

Based on the knowledge that MHC class II is upregulated on FLS in the RA joint we initially hypothesized that AIRE induces transcription of joint specific antigens in FLS. However, there was no enrichment of TRA genes and no induction of MHC class II genes in the AIRE high samples. Instead we found that AIRE mediates expression of IRGs in activated RA FLS, in particular a set of genes which have been associated with psoriasis skin lesions. This suggests a common inflammatory response in skin and the joint. Our data is supported by recent findings regarding keratin 17- and AIRE-dependent amplification of inflammatory and immune responses, in particular CXCL9, CXCL10, and CXCL11 expression, in skin undergoing acute inflammation or tumorigenesis (20). Furthermore, gene expression profiling of peripheral blood cells from healthy controls and RA patients in a Dutch study (30) revealed a significant up-regulation of IFN responsive genes in a subgroup of RA patients and six of these genes (*IFIT1, OAS2, MX1, IFI44L, OAS1*, and *MX2*) were also found in our dataset of top 65 AIRE regulated genes in FLS. In addition, response to anti-TNF treatment with infliximab in RA was associated with reduced expression of an IFN response gene set including *OAS1, MX2*, and *OAS2* (31) supporting a role for AIRE in the disease.

Within the interferon signature, there was a striking induction of chemokine genes also by AIRE in FLS, indicating that this master regulator promotes the recruitment of immune cells to the joint in arthritis. In particular, we found a strong AIRE-dependent induction and secretion of CXCL10 by TNF + IL-1β in RA FLS. CXCL10/IP10 is a chemoattractant for monocytes/macrophages, T lymphocytes, and dendritic cells and typically produced by fibroblasts and endothelial cells in response to INF-γ. A part from chemotaxis, CXCL10 have been reported to promote cancer invasion (32) and to increase invasive properties of RA FLS *in vitro* in a CXCR3-dependent autocrine fashion (33). The expression of CXCL10 is increased in synovial fluid and tissue in RA compared to controls (34) and serum levels of this chemokine correlate with disease activity (35, 36), highlighting its role in disease pathogenesis.

Our finding that AIRE induces a limited number of pro-inflammatory IRGs in FLS, as opposed to the large number of TRAs induced in mTECs, is intriguing. In thymic cells, it is believed that AIRE binds to the chromatin at multiple transcription start sites, guided by epigenetic marks and Sirt1, followed by recruitment of specific enzymes and transcription factors (17). The epigenetic landscape, and hence the accessibility of promotors, most likely differ in FLS, in particular in an inflammatory context, compared to mTECs. For example, IL-1β has been demonstrated to induce alterations in DNA methylation patterns in FLS (37) and might not only lead to AIRE expression, but also affect which set of genes AIRE induces. It is also possible that another set of transcription factors (interferon regulating factors?) are available or active for AIRE to interact with in activated FLS compared to in thymic cells.

Podoplanin is a pro-invasive glycoprotein with in large unknown function which is expressed on lymphatic endothelia and other specialized tissues but also up-regulated on tumor cells and on fibroblasts in reactive tissues such as synovitis in RA (22). We found synovial AIRE expression pre-dominantly in areas of high podoplanin expression. Likewise, AIRE and podoplanin were both induced in RA FLS *in vitro* by TNF + IL-1β in our RNA seq data set. This finding strengthens the link of AIRE to a subpopulation of activated aggressive FLS.

In conclusion, we demonstrate that the autoimmune regulator AIRE is expressed in activated FLS in the RA joint and induced *in vitro* by pro-inflammatory cytokines. Furthermore, we found that AIRE augments expression of an IFN-γ signature in RA FLS including a set of chemokine genes which have been associated with disease activity and with response to treatment in RA. Our findings support a novel extrathymic pro-inflammatory role of AIRE of importance for inflammatory conditions. Although further studies are required to fully understand the importance for AIRE in arthritis, our data supports a role for AIRE in peripheral effector cells in RA.

## Data Availability

The raw data supporting theconclusions of this manuscript will be made available by the authors upon request.

## Ethics Statement

This study was carried out in accordance with the recommendations of the Regional Ethical Review Board in Gothenburg with written informed consent from all subjects. All subjects gave written informed consent in accordance with the Declaration of Helsinki.

## Author Contributions

A-KE, BB, and GV recruited the patients and established the biobank. A-KE, OE, BB, GV, and CL designed the experiments and analyzed the data. BB, CL, GV, and A-KE conduced the experiments and acquired the data. A-KE directed the research and drafted the manuscript. OE, BB, CL, GV, and HC participated in writing the manuscript.

### Conflict of Interest Statement

The authors declare that the research was conducted in the absence of any commercial or financial relationships that could be construed as a potential conflict of interest.

## Supplementary Material

Table S1 TaqMan Gene expression assays used for qPCR.

Table S2 Antibodies and dilutions.

Table S3 RA susceptibility genes in activated RA FLS.

Table S4 Random gene sets in the TRA analysis.
