# Supplementary material for: The Rheumatoid Arthritis Risk Gene AIRE Is Induced by Cytokines in Fibroblast-Like Synoviocytes and Augments the Pro-inflammatory Response
Source: Front Immunol. 2019 Jun 18;10:1384. doi: 10.3389/fimmu.2019.01384 (PMC6591464; doi:10.3389/fimmu.2019.01384)
Supplement: Table S1 — TaqMan Gene expression assays used for qPCR. [file Data_Sheet_1.docx]

**Appendix A. Supplementary data**

*Table S1. TaqMan Gene Expression Assays used for qPCR*

|  |  |  |  |
| --- | --- | --- | --- |
| ***Gene Symbol*** | ***Gene Name*** | | ***Assay ID*** |
| *GAPDH* | glyceraldehyde-3-phosphate dehydrogenase | | Hs99999905_m1 |
| *AIRE* | autoimmune regulator | | Hs00230829_m1 |
| *PADI4* | peptidyl arginine deiminase 4 | | Hs01057483_m1 |
| *INS* | insulin | | Hs00355773_m1 |
| *CXCL10* | C-X-C motif chemokine ligand 10 | | Hs00171042_m1 |
| *CCL8* | C-C motif chemokine ligand 8 | | Hs04187715_m1 |

|  |  |  |  |
| --- | --- | --- | --- |
| ***Antibody*** | ***Clone/***  ***catalogue no*** | ***Company*** | ***Dilution*** |
| Goat anti-human AIRE | D-17 | Santa Cruz Biotechnology | 100 |
| Goat anti-human AIRE | Ab78065 | Abcam | 200 |
| Mouse anti-human podoplanin | D2-40 | AbD Serotec | 20 |
| Rabbit anti-mouse Alexa Fluor 488 | A11059 | Life Technologies | 500 |
| Donkey anti-goat Alexa Fluor 555 | A21432 | Life Technologies | 500 |
| Rat anti-human AIRE-biotin | TM-724 | eBioscience | 100 |
| Rat IgG2a-biotin | eBR2a | eBioscience | 100 |
|  |  |  |  |

*Table S2. Antibodies and dilutions*

*Table S3. Genes overlapping between the datasets “DE genes by TNFα + IL-1β” and “RA risk genes” (*).*

|  |  |  |
| --- | --- | --- |
| ***Gene Symbol*** | ***Gene Name*** | |
| *AFF3* | AF4/FMR2 family member 3 | |
| *AIRE* | autoimmune regulator | |
| *ANXA3* | annexin A3 | |
| *B3GNT2* | UDP-GlcNAc:betaGal beta-1,3-N-acetylglucosaminyltransferase 2 | |
| *CD226* | CD226 molecule | |
| *CD83* | CD83 molecule | |
| *CFLAR/CASP8* | CASP8 and FADD like apoptosis regulator / caspase 8 | |
| *CSF2* | colony stimulating factor 2 | |
| *HLA-B* | major histocompatibility complex, class I, B | |
| *IFNGR2* | interferon gamma receptor 2 (interferon gamma transducer 1) | |
| *LBH* | limb bud and heart development | |
| *MTF1/INPP5B* | metal-regulatory transcription factor 1 / inositol polyphosphate-5-phosphatase B | |
| *NFKBIE* | NFKB inhibitor epsilon | |
| *PRDM1* | PR domain 1 | |
| *PTPN2* | tyrosine-protein phosphatase non-receptor type 2 | |
| *RBPJ* | recombination signal binding protein for immunoglobulin kappa J region | |
| *RCAN1* | regulator of calcineurin 1 | |
| *REL* | REL proto-oncogene, NF-KB subunit | |
| *RUNX1* | runt-related transcription factor 1 | |
| *TLE3* | transducin-like enhancer of split 3 | |
| *TNFAIP3* | TNF alpha induced protein 3 | |
| *TNFRSF9* | tumor necrosis factor receptor superfamily member 9 | |
| *TRAF1* | TNF receptor-associated factor 1 | |
| *TXNDC11* | thioredoxin domain containing 11 | |
|  |  | |

** Yarwood A, Huiziga T and Worthington J. The genetics of rheumatoid arthritis: risk and protection in different stages of the evolution of RA. Rheumalogy. 2016 Feb;55(2):199-209*
